# Bufalin suppresses tumour microenvironment-mediated angiogenesis by inhibiting the STAT3 signalling pathway

**DOI:** 10.1186/s12967-021-03058-z

**Published:** 2021-09-08

**Authors:** Kai Fang, Yueping Zhan, Ruiqiu Zhu, Yuqian Wang, Chengqi Wu, Min Sun, Yanyan Qiu, Zeting Yuan, Xin Liang, Peihao Yin, Ke Xu

**Affiliations:** 1 Putuo Hospital, Shanghai University of Traditional Chinese Medicine, Shanghai, 200062 China; 2 Institute of Translational Medicine, Shanghai University, Shanghai, 200444 China; 3 Interventional Cancer Institute of Chinese Integrative Medicine, Shanghai University of Traditional Chinese Medicine, Shanghai, 200062 China; 4 State Key Laboratory of Bioreactor Engineering & Shanghai Key Laboratory of New Drug Design, School of Pharmacy, East China University of Science and Technology, 130 Meilong Rd, Shanghai, 200237 China; 5 Shanghai Putuo Central School of Clinical Medicine, Anhui Medical University, Hefei, 230032 China; 6 Wenzhou Institute of Shanghai University, Wenzhou, 325000 China

**Keywords:** Angiogenesis, Bufalin, Colorectal cancer, STAT3, Tumour microenvironment

## Abstract

**Background:**

Antiangiogenic therapy has increasingly become an important strategy for the treatment of colorectal cancer. Recent studies have shown that the tumour microenvironment (TME) promotes tumour angiogenesis. Bufalin is an active antitumour compound whose efficacy has been indicated by previous studies. However, there are very few studies on the antiangiogenic effects of bufalin.

**Methods:**

Herein, human umbilical vein endothelial cell (HUVEC) tube formation, migration and adhesion tests were used to assess angiogenesis in vitro. Western blotting and quantitative PCR were used to detect relevant protein levels and mRNA expression levels. A subcutaneous xenograft tumour model and a hepatic metastasis model were established in mice to investigate the influence of bufalin on angiogenesis mediated by the TME in vivo.

**Results:**

We found that angiogenesis mediated by cells in the TME was significantly inhibited in the presence of bufalin. The results demonstrated that the proangiogenic genes in HUVECs, such as VEGF, PDGFA, E-selectin and P-selectin, were downregulated by bufalin and that this downregulation was mediated by inhibition of the STAT3 pathway. Overexpression of STAT3 reversed the inhibitory effects of bufalin on angiogenesis. Furthermore, there was little reduction in angiogenesis when bufalin directly acted on the cells in the tumour microenvironment.

**Conclusion:**

Our findings demonstrate that bufalin suppresses tumour microenvironment-mediated angiogenesis by inhibiting the STAT3 signalling pathway in vascular endothelial cells, revealing that bufalin may be used as a new antiangiogenic adjuvant therapy medicine to treat colorectal cancer.

**Supplementary Information:**

The online version contains supplementary material available at 10.1186/s12967-021-03058-z.

## Background

Colorectal cancer (CRC) is the third most common cancer worldwide and accounts for 9% of all cancer-related deaths [1], and its incidence is increasing. It is now widely accepted that angiogenesis plays a key role in tumour development, progression and metastasis [2]. Abnormal angiogenesis is a hallmark of solid tumours [3]. The concept of antiangiogenic therapy arose from the seminal observations by Judah Folkman and colleagues. It has been reported that antiangiogenic therapy can effectively improve the survival rate of CRC patients, which indicates that inhibiting tumour angiogenesis is a potential method for treating CRC [4–7].

Many studies have demonstrated that the TME including the cancer-associated fibroblasts (CAFs) and tumour-associated macrophages (TAMs) contained within it, promotes tumour angiogenesis [8–10]. The tumour microenvironment, which is also termed the tumour mesenchyme or tumour stroma, includes CAFs, TAMs, blood vessels and extracellular matrix and substantially influences the initiation, growth and dissemination of CRC [3]. Signal transducer and activator of transcription 3 (STAT3) belongs to a family of transcription factors that regulate the expression of genes involved in the pathogenesis of many human malignancies [11, 12]. It was also reported that the TME activates STAT3 signalling in human umbilical vein endothelial cells [13], and that the tumour microenvironment may affect angiogenesis through the STAT3 signalling pathway. In summary, the STAT3 pathway in blood vessels may become a target for the treatment of angiogenesis.

**Fig. 1:**
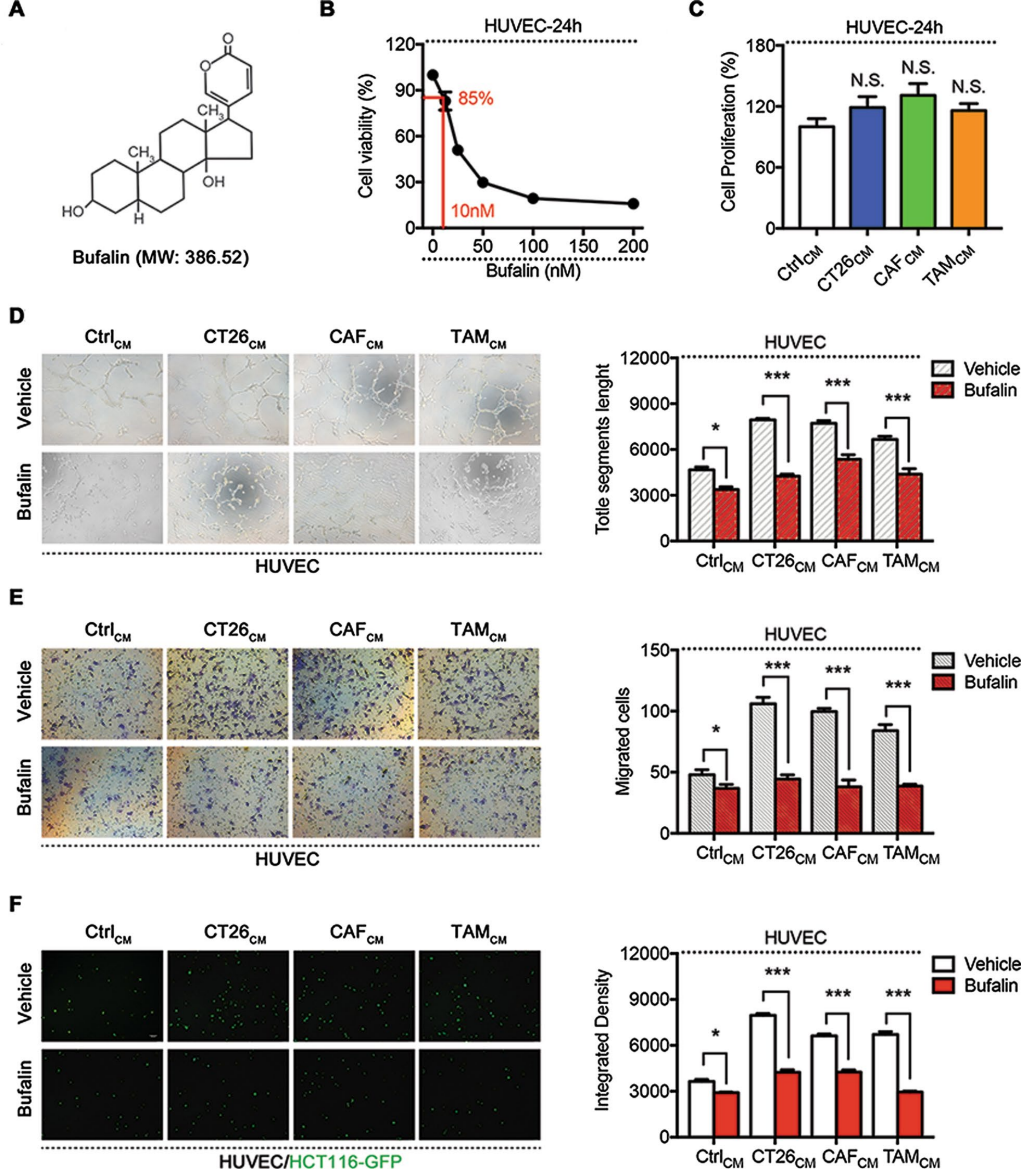
Bufalin suppresses angiogenesis induced by cells in the tumour microenvironment cells. **a** Molecular structure of BU. **b** Cell viability of HUVECs after treated with BU for 24 h. **c** Cell proliferation of HUVECs after treated with different TME-CMs for 24 h. The effect of bufalin (BU) on the tube formation (**d**), migration (**e**) and adhesion (**f**) of HUVECs in response to different TME-CMs. *P < 0.05, **P < 0.01, ***P < 0.001. Data are shown as mean s.e.m. BU, bufalin

Bufalin (BU), the major bioactive component isolated from toad venom (Fig. 1a), has been confirmed to be a potent antitumour drug due to its effects on tumour cell apoptosis, metastasis and proliferation [14, 15]. In addition, bufalin inhibits angiogenesis, and it was reported that the antiangiogenic effects of sorafenib were significantly increased when used in combination with bufalin by targeting AKT/VEGF in HUVECs [16]. Wu et al. showed that bufalin enhanced cytocidal effects by targeting the STAT3 pathway [17].

Previously, we performed numerous studies on bufalin for the treatment of colorectal cancer [14, 18, 19]. Here, we found that bufalin could reverse the proangiogenic effects mediated by the TME. In the present study, we used in vitro angiogenesis-related experiments, an in vivo subcutaneous tumour model and an in vivo liver metastasis model to demonstrate that bufalin can inhibit TME-mediated STAT3 activation in endothelial cells to reduce angiogenesis, identifying a new mechanism of action of bufalin in the treatment of CRC.

## Materials and methods

### Cell culture

Cells were cultured in a humidified incubator with an atmosphere of 5% CO_2_ at 37 °C under normal oxygen conditions. Human umbilical vein endothelial cells (HUVECs; #8000, ScienCell, USA) were grown in endothelial cell medium (ECM; #1001, ScienCell, USA), and only early passages (< p6) were used. CT26 cells were obtained from the Cell Bank of the Chinese Academy of Sciences and were cultured in RPMI-1640 containing 10% FBS and 1% penicillin/streptomycin. The STAT3 overexpression plasmid was purchased from GeneChem (Shanghai, CN).

### Conditioned medium (CM) preparation

CT26 cells were confirmed by morphological observation (Additional file 1: Fig. S1a) and WB (Additional file 1: Fig. S1b). The tumour cell supernatant polarized the M0 macrophages, which was confirmed by morphological observation and flow cytometry (Additional file 1: Fig. S1c, d). When TAMs, CAFs or CT26 cells grew to 80% confluence, the medium was replaced with FBS-free medium. After 48 h of treatment, cell suspensions were collected as the CM. The CM was collected after high-speed centrifugation and then filtered through a 0.22 μm microporous membrane and stored at − 20 °C. In the same way, the cells were treated with 10 nM bufalin for 24 h and then treated as described above to obtain the relevant conditioned medium after drug treatment.

### Tube formation assay

HUVECs were incubated with conditioned medium for 24 h before the tube formation assay. Matrigel (BD, #356234, USA) was thawed overnight at 4 °C, and 50 μl of Matrigel was spread in a 96-well plate and polymerized at 37 °C for 30 min. Next, 3 × 10^4^ HUVECs in 50 μl of ECM were seeded into each well for incubation at 37 °C in 5% CO_2_ for 4 h. HUVECs were photographed under a microscope.

### Cell migration assay

HUVECs were incubated with conditioned medium for 24 h before the migration assay. HUVECs (3 × 10^4^) in 300 μl of serum-free ECM were added to the upper chamber (8.0 μm pore size, #353,097, FALCON, USA), and allowed to migrate towards 700 μl of complete ECM. The unmigrated cells were removed from the upper part of the Transwell chamber with a cotton swab after 6 h of incubation, and the insert was fixed with 4% paraformaldehyde for 10 min at room temperature. The Transwell inserts were stained with 500 μl of 0.03% crystal violet solution for 30 min at 37 °C. Then, the insert was immersed in PBS and washed three times with PBS for 5 min each time, and then dried and photographed under a microscope.

### Adhesion assay

HUVECs were seeded in a 24-well plate and then treated with conditioned medium for 24 h. HCT116-GFP cells (2 × 10^4^) in 300 μl of serum-free ECM were coincubated with the treated HUVECs for incubation at 37 °C for 2 h. Cells were washed with PBS three times and then photographed under a fluorescence microscope.

### Quantitative PCR

Total RNA was extracted from HUVECs using TRIzol (Invitrogen). The concentration of total RNA was quantified by measuring the absorbance at 260 nm. For SYBR Green-based quantitative PCR amplification, the reaction was carried out in a volume of 20 μl (Applied Biosystems). The 2^−ΔΔCt^ method was used to determine the relative expression level of each cell line in each group.

The primer sequences were as follows: VEGF, 5′-TTGCTGCTCTACCTCCAC-3′ and 5′-AATGCTTTCTCCGCTCTG-3′; PDGFA, 5′-AGGCGTCCAGGCAGGTGATC-3′ and 5′-GCTTCTTCCTCGGTGCGTTCC-3′; E-selectin, 5′-ATGTTCAAGCCTGGCAGTTCCG-3′ and 5′-GCAGAGCCATTGAGCGTCCATC-3′; and P-selectin, 5′-CGCTCTGGACCAACCCTGTTTC-3′ and 5′-CTCCTGGCTTCTGTGGCTTGTG-3′.

### Western blot (WB)

The proteins were separated by SDS–PAGE. After incubation with blocking solution (5% nonfat dry milk in PBST) for 1 h at room temperature, the membranes were exposed to primary antibodies overnight at 4 °C (VEGF, Abcam, ab46154, rabbit IgG), (P-STAT3, CST, 4113S, mouse IgG), (STAT3, CST, 9139S, mouse IgG), (E-cadherin, CST, 3195S, rabbit IgG), (Vimentin, CST, 5741S, rabbit IgG), (α-SMA, Sigma, A2547, mouse IgG), and (β-actin, Abcam, ab6276, mouse IgG). The membranes were further probed with horseradish peroxidase-conjugated anti-rabbit/mouse IgG antibody (CST, 1:5000). ImageJ software was used to quantify the protein bands.

### In vivo* xenograft model*

To determine the antiangiogenic activity of bufalin in vivo, CT26-LUC cells (2 × 10^6^) were injected subcutaneously or into the spleens of male BALB/c mice (6 weeks old). 1 week after injection, bufalin (1 mg/kg) was administered by intraperitoneal (i.p.) injection once every other day for 21 days (flank) or 14 days (spleen). The vehicle group was treated with normal saline. Subcutaneous tumour sizes were measured every 3 days, and live imaging was performed once a week after treatment. The estimated tumour volume (V) was calculated by the Formula V = W^2^ × L × 0.5, where W represents the largest tumour diameter in centimetres and L represents the next largest tumour diameter. Tumour-bearing mice were sacrificed after 21 or 14 days of treatment, and the tumour tissues, spleens and livers were harvested, weighed, and immediately fixed in formalin for follow-up experiments.

All experiments conformed to the ethical principles of animal experimentation stipulated by the Institutional Animal Care and Use Committee of Putuo Hospital, Shanghai University of Traditional Chinese Medicine, China.

### Histopathological assay

Tissues were harvested after the animals were sacrificed. The histopathological assay procedure was carried out by conventional haematoxylin–eosin (H&E) staining in accordance with standard techniques.

### Immunofluorescence

HUVECs (2 × 10^4^) were seeded and cultured overnight on microscope coverslips (Thermo Fisher Scientific, Waltham, MA, USA). After treatment with CM with or without bufalin for 24 h as described previously, the cells were washed with PBS twice, fixed in methanol for 15 min, permeabilized with 0.2% Triton X-100 (Beyotime, Shanghai, China)/PBS for 5 min and blocked with 5% BSA for 1 h at room temperature. The coverslips were incubated with primary antibodies at 4 °C overnight (p-STAT3, CST, 4113S, mouse IgG) and (CD31, Abcam, ab28364, rabbit IgG) and then incubated with secondary antibodies for 2 h at 37 °C protected from light (goat anti-mouse IgG H&L (Alexa Fluor® 488), Abcam, ab150113) and (goat anti-rabbit IgG H&L (Alexa Fluor® 594), Abcam, ab150080). Nuclear localization was assessed with 4′,6-diamidino-2-phenylindole (DAPI; Beyotime, Shanghai, China).

Tissue sections were permeabilized with cold methanol for 5 min and incubated with 5% BSA in PBS for 1 h. Primary antibodies were applied in blocking buffer and incubated overnight at 4 °C. Dye-conjugated secondary antibodies were added to the blocking buffer and incubated for 2 h. Nuclei were stained with 4′,6-diamidino-2-phenylindole (DAPI; Beyotime, Shanghai, China). Images were acquired using a Zeiss LSM880 confocal microscope at the same voltage level and analysed using ZEN Software.

### Immunohistochemistry (IHC)

The tissues were fixed in 10% formalin, embedded in paraffin, and then sectioned (5 mm thick). IHC of CD31 was performed as follows. The slides were dewaxed and incubated with a 3% aqueous solution of H_2_O_2_ for 10 min to quench endogenous peroxidase activity. The heat-induced antigen recovery method was used to detect the antigen. Tissues were incubated with 5% BSA for 30 min at room temperature and then incubated with the primary antibody in PBS at 4 °C overnight (CD31, Abcam, ab28364, rabbit IgG) and (Ki67, Abcam, ab15580, rabbit IgG). The appropriate secondary antibody was used to apply the indirect avidin–biotin-peroxidase method at room temperature for 30 min. An EnVision (K4007, Dako) signal enhancement system was used to develop the bound antibodies. The sections were stained with Harris haematoxylin, dehydrated and fixed. For quantification, 30 random images (400 ×) were captured with a microscope (Leica, Wetzlar, Germany).

### ELISA

VEGF in HUVEC culture supernatants was evaluated by a Human VEGF Quantikine ELISA Kit (R&D, Minnesota, USA) according to the manufacturer’s protocol. VEGF in serum was evaluated by a Mouse VEGF Quantikine ELISA Kit (R&D, Minnesota, USA) according to the manufacturer’s protocol.

### Statistical analysis

All statistical analyses were performed using Excel 2016, GraphPad Prism 8, and R (v.8.0.1) software. Each experimental value is expressed as the mean ± SD. An unpaired t-test was performed for comparisons between two groups and one-way analysis of variance (ANOVA) was applied for pairwise comparisons among multiple groups with Tukey’s post hoc test, and significance was accepted at *p < 0.05, **p < 0.01 and ***p < 0.001.

## Results

### Bufalin suppresses angiogenesis induced by cells in the tumour microenvironment

To clarify the effects of bufalin on angiogenesis caused by TME cells, we collected the supernatants of CT26 cells, CAFs and TAMs as TME-conditioned media (CMs). Furthermore, we selected a low concentration (IC_15_, 10 nM) of bufalin to treat HUVECs for 24 h (Fig. 1b). We examined the mechanism by which bufalin inhibits angiogenesis and affects the occurrence and development of colorectal cancer. The cell proliferation experiment showed that the TME had no significant effect on HUVEC proliferation after treatment with TME-CMs (CT26-CM, CAF-CM and TAM-CM) for 24 h (Fig. 1c). The effects of bufalin on TME-mediated angiogenesis was determined by HUVEC tube formation, migration and adhesion experiments, and the results showed that bufalin inhibited angiogenesis induced by TME cells (Fig. 1d–f).

### Bufalin suppresses TME-mediated angiogenesis by inhibiting angiogenic factors

**Fig. 2:**
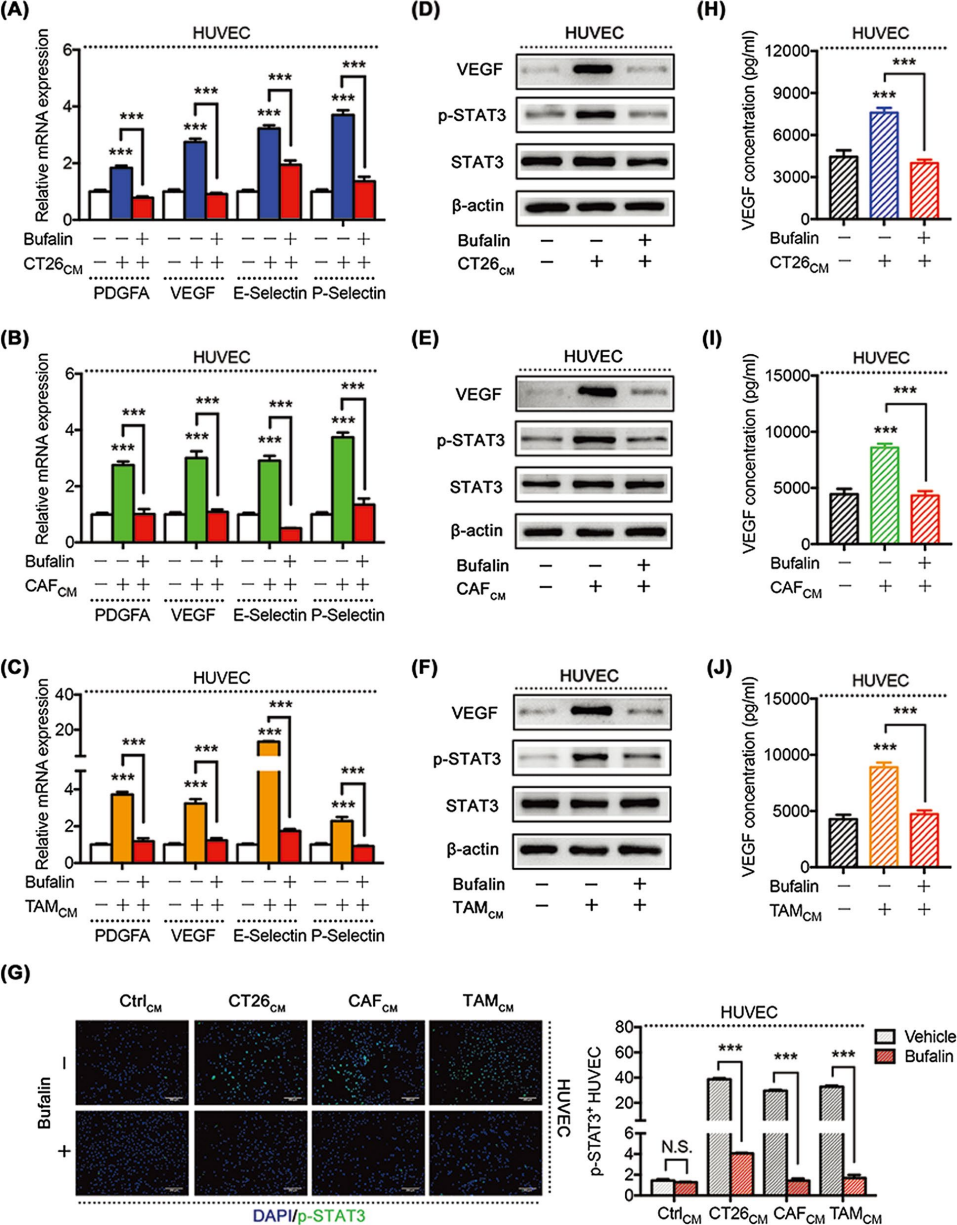
Bufalin suppresses TME-mediated angiogenesis by inhibiting angiogenic factors. Relative PDGFA, VEGF, E-selectin and P-selectin mRNA expression in HUVECs after treatment with CT26-CM (**a**), CAF-CM (**b**) or TMA-CM (**c**) with or without bufalin (BU). **d–f** WB showing the protein expression of VEGF, p-STAT3 and STAT3 in HUVECs treated with different TME-CMs in the presence or absence of BU, with total β-actin as a control. **g–i** The concentration of VEGFA in HUVECs supernatant after treatment with different TME-CMs in the presence or absence of BU. **j** Immunofluorescence analysis showing p-STAT3^+^ HUVECs after treatment with different TME-CMs in the presence or absence of BU. *P < 0.05, **P < 0.01, ***P < 0.001. Data are shown as mean s.e.m. BU, bufalin

To determine the mechanism by which bufalin inhibits TME-mediated angiogenesis, we observed the expression of the angiogenesis-related and migratory factors VEGF and PDGFA and the vascular adhesion genes E-selectin and P-selectin in HUVECs treated with or without bufalin in combination with TME-CMs by quantitative PCR. The results showed that bufalin could significantly downregulate the expression of these genes in HUVECs that were increased by the TME (Fig. 2a–c).

Previous studies have shown that the STAT3 signalling pathway plays a critical role in angiogenesis by inducing angiogenic factors, such as VEGFs, PDGFs and other angiogenic factors [20, 21]. In our study, the WB experiment showed that bufalin could directly decease STAT3 phosphorylation in HUVECs activated by the TME (Fig. 2d–f), and the results of the immunofluorescence experiment confirmed these findings (Fig. 2g). In addition, we found that bufalin could inhibit VEGF expression by WB (Fig. 2d–f) and ELISA (Fig. 2h–j). Taken together, these results suggest that bufalin could decrease STAT3 phosphorylation to downregulate angiogenic factor expression induced by the TME in vascular endothelial cells.

### Bufalin suppresses TME-mediated angiogenesis by the STAT3 signalling pathway

**Fig. 3:**
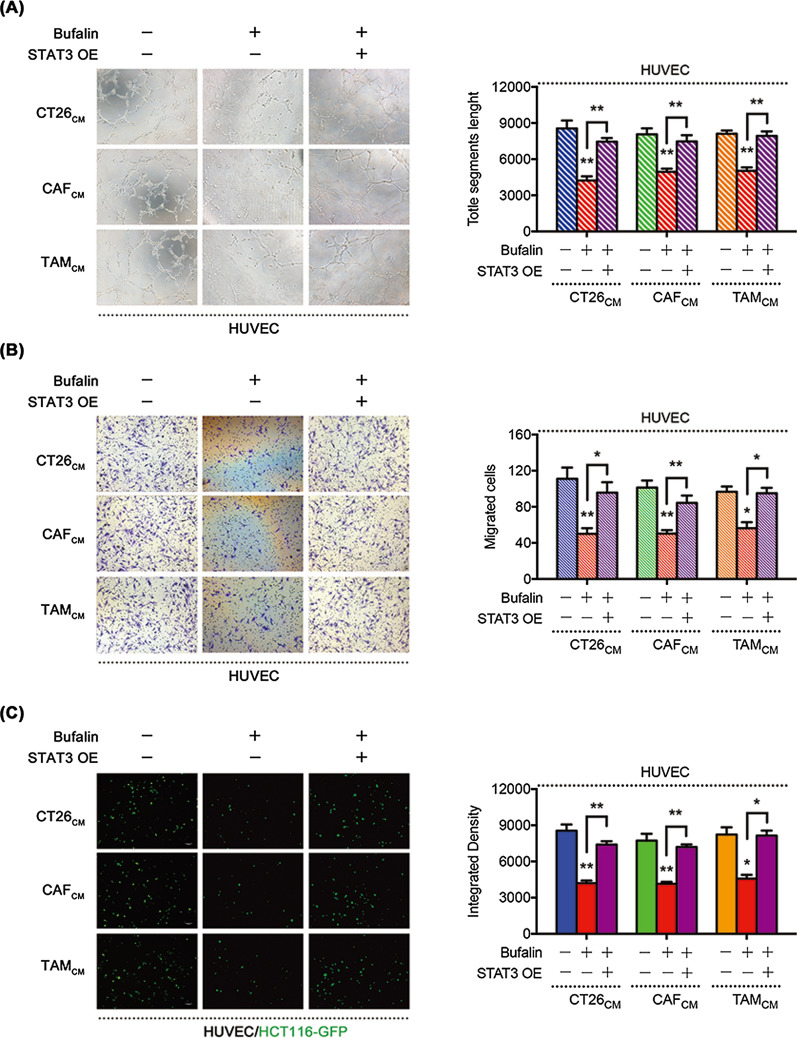
Bufalin suppresses TME-mediated angiogenesis by the STAT3 signalling pathway. Tube formation (**a**), migration (**b**) and adhesion (**c**) of HUVECs after treatment with TME-CMs and BU with or without the STAT3-OE plasmid. *P < 0.05, **P < 0.01, ***P < 0.001. Data are shown as mean s.e.m. BU, bufalin

To investigate whether the STAT3 signalling pathway is a key factor by which bufalin inhibits TME-mediated angiogenesis, we constructed a STAT3-overexpression (STAT3-OE) plasmid. STAT3 expression was confirmed by WB (Additional file 2: Fig. S2a) and qPCR (Additional file 2: Fig. S2b). We found that the STAT3-OE plasmid prevented bufalin-mediated inhibition of HUVEC tube formation (Fig. 3a), migration (Fig. 3b) and adhesion (Fig. 3c) induced by the TME. These results suggest that bufalin inhibits TME-induced angiogenesis through the STAT3 signalling pathway and that STAT3 plays an important role in this process.

### Bufalin suppresses TME-mediated angiogenesis by directly affecting vascular endothelial cells but not altering tumour microenvironment cells

**Fig. 4:**
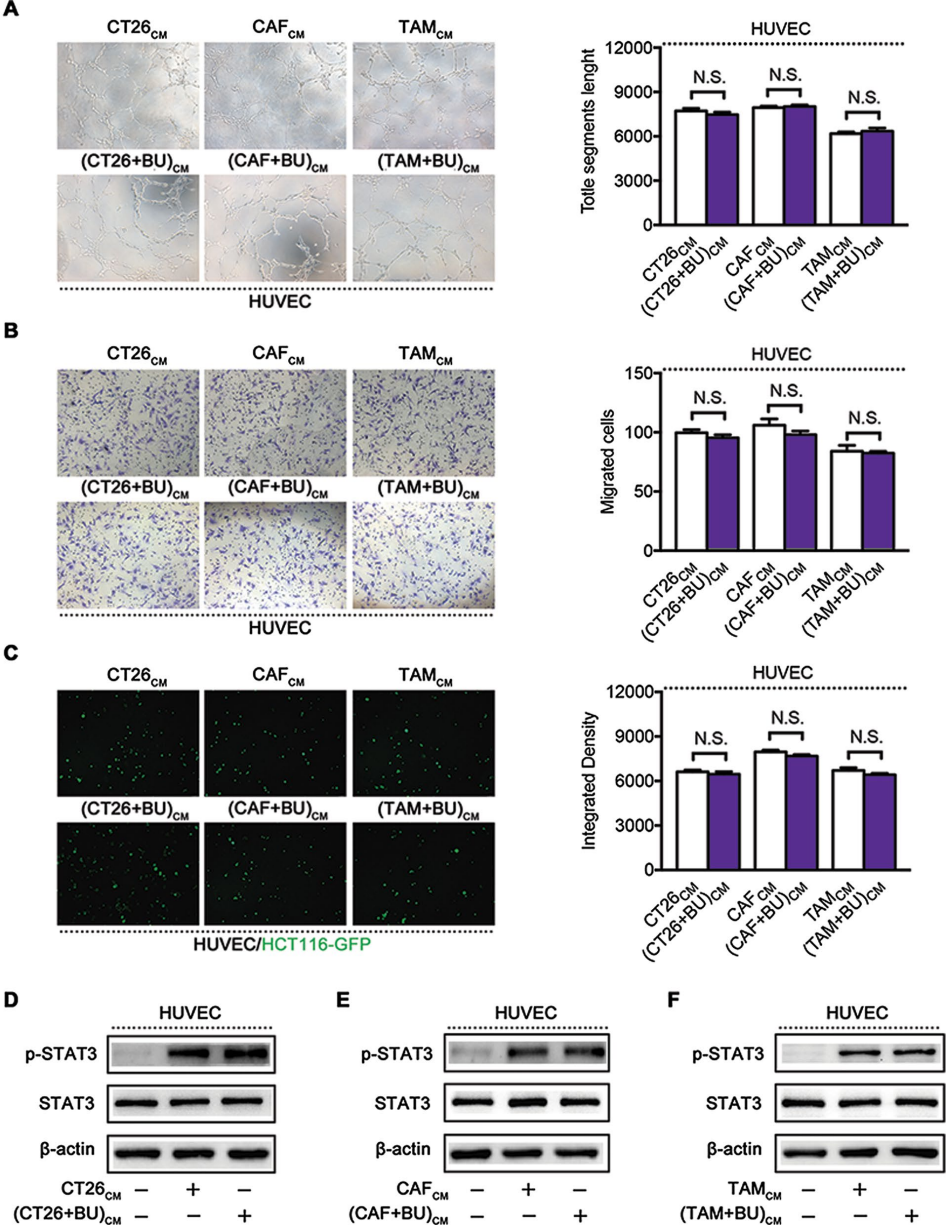
Bufalin suppresses TME-mediated angiogenesis by directly affecting vascular endothelial cells. Tube formation (**a**), migration (**b**), and adhesion (**c**) of HUVECs treated with TME-CMs and TME + BU-CMs for 24 h. **d–f** WB showing the protein expression of p-STAT3 and STAT3 in HUVECs treated with different TME-CMs and TME + BU-CMs, membranes were stripped and re-probed with total β-actin as a control. N.S. indicates no significant difference between the two groups, P > 0.05. Data are shown as mean s.e.m. BU, bufalin

Next, we determined whether bufalin also indirectly affects endothelial cells by altering TME cells. We first treated TME cells (CT26 cells, CAFs and TAMs) with 10 nM bufalin for 24 h, replaced the medium with serum-free medium and collected the cell supernatant after an additional 24 h of incubation for use as (TME + BU)-CM, which was then used to treat HUVECs for 24 h. Then, HUVEC tube formation (Fig. 4a), migration (Fig. 4b) and adhesion (Fig. 4c) were analysed. The results showed that bufalin did not affect vascular endothelial cells by affecting TME cells. The WB results also proved that there were no significant differences in the increase in phosphorylated STAT3 levels in HUVECs induced by (TME + BU)-CMs and TME-CMs (Fig. 4d–f). Taken together, these results suggest that bufalin suppresses TME-mediated angiogenesis by directly affecting vascular endothelial cells but not altering TME cells.

### *Bufalin inhibits CRC cell growth *via* an antiangiogenic mechanism *in vivo

**Fig. 5:**
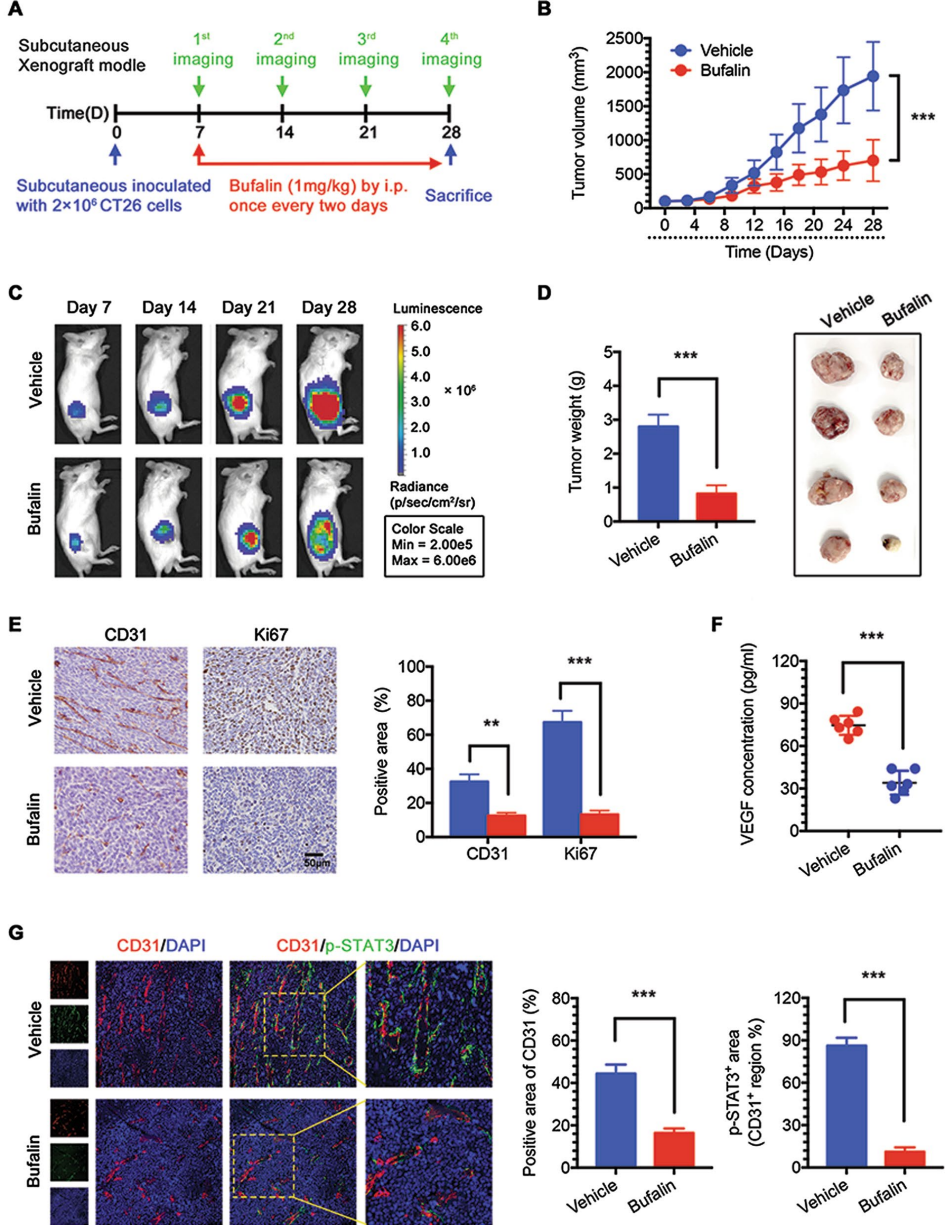
Bufalin inhibits CRC cell growth via an antiangiogenic mechanism in vivo. **a** Scheme and schedule of imaging and treatments. **b** Tumour volumes from day 0 to day 28. **c** Tumour growth was visualized by an in vivo imaging system from day 7 to day 28. **d** Tumour weights and images. **e** IHC analysis of CD31 and Ki67 in tumours. **f** VEGF expression level in serum. **g** Immunofluorescence analysis of CD31 and p-STAT3 in tumours. **P < 0.01, ***P < 0.001. Data are shown as mean s.e.m. IHC, immunohistochemistry

To determine the effects of bufalin in vivo, a CRC cell xenograft model was established by using CT26 cells expressing luciferase (CT26-LUC). The schedule is shown in Fig. 5a. All mice started treatment 1 week after xenotransplantation. The volumes of the subcutaneous tumours (Fig. 5b) and in vivo imaging (Fig. 5c, Additional file 3: Fig. S3b) were recorded during the treatment cycle (3 weeks). Subcutaneous tumours in the two groups of mice were weighed and photographed (Fig. 5d). The results showed that bufalin significantly inhibited tumour growth compared with vehicle without affecting animal body weight (Additional file 3: Fig. S3a), suggesting that bufalin had no serious toxic effects on the body. To further confirm the antiangiogenic effects obtained in vitro, we used IHC to evaluate the expression of CD31 and Ki67 in the tumours. The results of IHC showed a reduction in proliferation and subcutaneous tumour blood vessels in the bufalin group (Fig. 5e). Next, we measured the serum VEGF levels in the two groups of mice by ELISA, and the results showed that the serum VEGF levels in the bufalin group were significantly lower than those in the vehicle group (Fig. 5f). Moreover, we examined the activation of vascular STAT3 in solid tumours. The immunofluorescence results showed that the number of blood vessels in subcutaneous tumours and the proportion of activated STAT3-positive blood vessels in the bufalin group were significantly lower than those in the vehicle group (Fig. 5g). These data indicate that bufalin inhibited angiogenesis by targeting the activation of STAT3 in tumour blood vessels, thereby inhibiting tumour growth in a CRC cell xenograft model.

### *Bufalin inhibits CRC cell metastasis *via* an antiangiogenic mechanism *in vivo

**Fig. 6:**
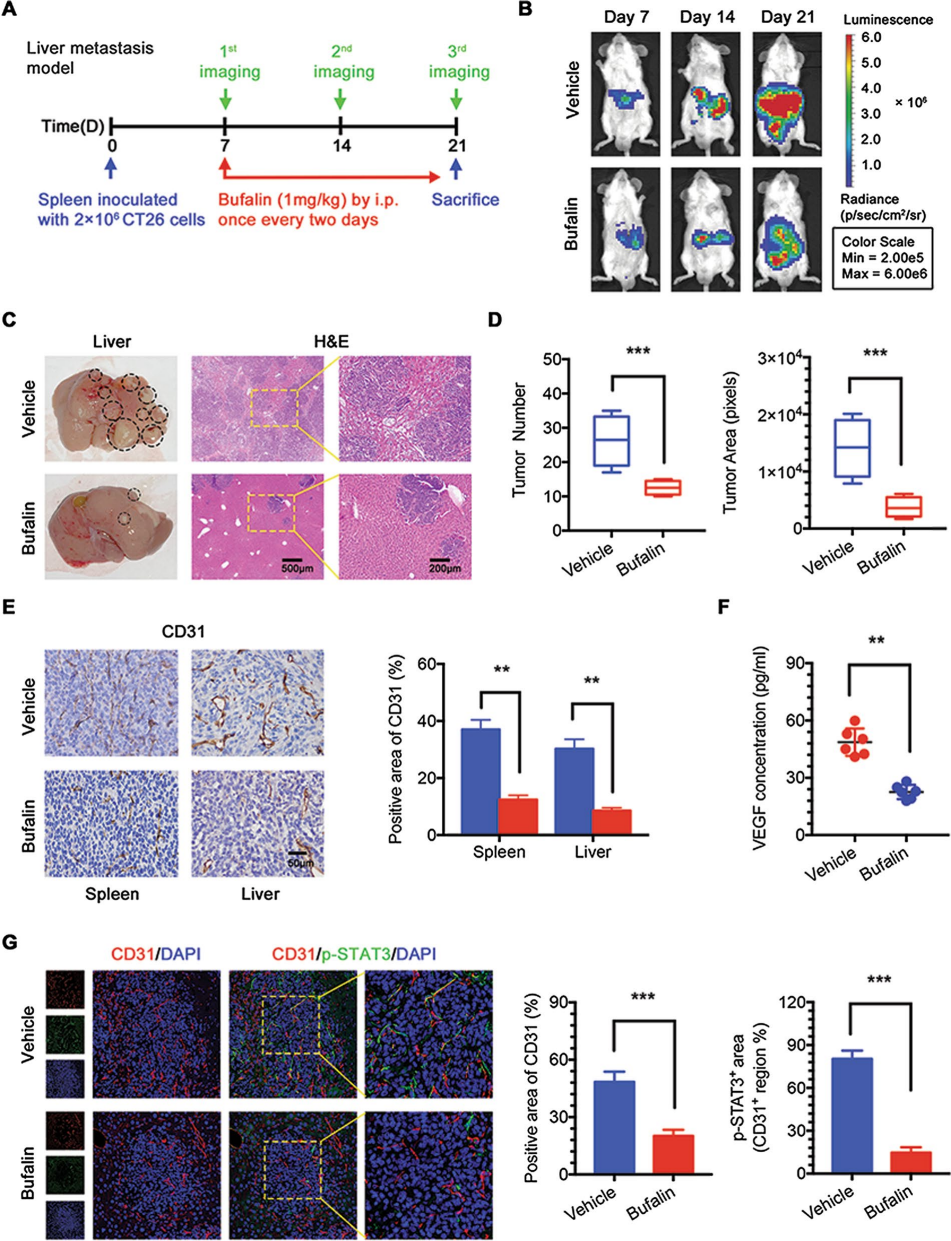
Bufalin inhibits CRC cell metastasis via an antiangiogenic mechanism in vivo. **a** Scheme and schedule of imaging and treatments. **b** Tumour metastasis was visualized by an in vivo imaging system from day 7 to day 21. **c** Representative images of liver and H&E-stained liver tissue. **d** Tumour number and area in liver. **e** IHC analysis of CD31 in spleen and liver tissue. **f** VEGF expression level in serum. **g** Immunofluorescence analysis of CD31 and p-STAT3 in liver. **P < 0.01, ***P < 0.001. Data are shown as mean s.e.m. IHC, immunohistochemistry

Cancer metastasis remains a major challenge for the successful management of malignant diseases. The liver is the main site of metastatic disease and a major cause of death from colorectal cancer [22]. To investigate the antiangiogenic effects of bufalin on the inhibition of CRC metastasis in vivo, we established a liver metastasis model (Fig. 6a), and the progression of tumour metastasis was observed by an in vivo imaging system from Day 7 to Day 21 (Fig. 6b, Additional file 4: Fig. S4b). The in vivo imaging results showed that after 1 week of bufalin treatment, bufalin began to inhibit liver metastasis compared to the vehicle group, and until the mice were sacrificed on Day 21, bufalin significantly inhibited liver metastases by more than 50% compared to vehicle treatment without affecting animal body weight (Additional file 4: Fig. S4a). Metastatic foci of considerable sizes were visible in the livers of mice treated with vehicle. Haematoxylin–eosin–stained liver sections were examined under a microscope, and as expected, the formation of metastases in the liver was reduced by approximately 80% after bufalin treatment (Fig. 6c, d). The IHC results showed a reduction in the number of blood vessels in the spleen and liver metastases in the bufalin group (Fig. 6e). Similarly, mice treated with bufalin had significantly lower serum VEGF levels than mice treated with vehicle (Fig. 6f). Consistent with the subcutaneous tumours, the immunofluorescence results showed that the number of blood vessels in the liver tumours and the number of p-STAT3-positive blood vessels significantly decreased after bufalin treatment (Fig. 6g). Interestingly, STAT3 in endothelial cells was activated only at the tumour site, while it was rarely activated in normal liver tissues. This result further suggests that bufalin inhibits liver metastasis by targeting STAT3 in tumour blood vessels not only in primary tumours but also in metastatic tumours (Fig. 6g).

## Discussion

Accumulating evidence has verified that angiogenesis plays a critical role in tumour progression and that inhibiting angiogenesis is a promising strategy for tumour treatment [23, 24]. Angiogenic programming in neoplastic tissue is a multidimensional process regulated by tumour cells in conjunction with various tumour-associated stromal cells, as well as the TME [10, 25–27]. We found that bufalin could reverse angiogenesis mediated by the TME.

Antiangiogenic therapy is an important strategy for the treatment of CRC. Previous studies have reported that bufalin can synergistically enhance the antiangiogenic effects of sorafenib via AKT/VEGF signaling [16]. We found that bufalin could inhibit the tube formation, adhesion and migration of HUVECs mediated by CAFs, TAMs and tumour cells by inhibiting the activation of HUVEC STAT3 and thereby decreasing the expression of VEGF, PDGFA, E-selectin, and P-selectin. Similarly, we established a subcutaneous tumour model and a liver metastasis model in vivo, and we found that bufalin inhibited the growth and metastasis of CRC by significantly reducing the number of blood vessels and amount of STAT3 phosphorylation in vascular endothelial cells. In addition, the serum concentration of VEGF after bufalin treatment was significantly reduced. These findings indicate that bufalin targets the STAT3 signalling pathway to reduce TME-mediated angiogenesis.

**Fig. 7:**
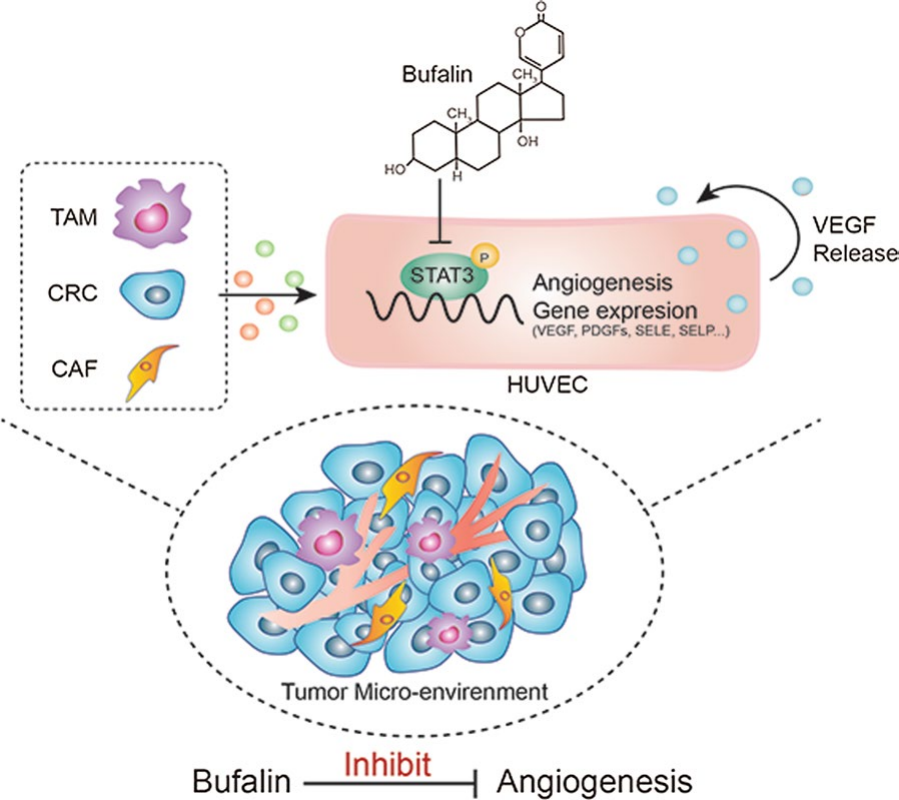
The mechanism of BU inhibiting tumor microenvironment-mediated angiogenesis

STAT3 is a transcription factor that regulates various kinds of cellular events, including differentiation, apoptosis and proliferation [28]. Previous studies have shown that STAT3 activation promotes tumour angiogenesis by increasing VEGF expression [29, 30]. Intercellular communication between the TME and vascular endothelial cells is promoted by STAT3 [31]. Notably, STAT3 is an important target of bufalin, and bufalin can inhibit STAT3 activity. Moreover, we found that STAT3 overexpression could reverse the inhibitory effects of bufalin on angiogenesis. Collectively, we propose a model in which bufalin reduces the expression of angiogenic genes by inhibiting the phosphorylation of STAT3 on endothelial cells, thereby antagonizing the proangiogenic effects of the tumour microenvironment (Fig. 7).

More interestingly, we found that bufalin could suppress TME-mediated angiogenesis. Furthermore, we treated CAFs, TAMs and tumour cells with bufalin and then used the conditioned cell supernatants to treat HUVECs and found no effects on tube formation, migration or adhesion, which ruled out the direct effects of bufalin on TME cells. Notably, compared to other previous antitumour studies on bufalin [32, 33], we chose a lower concentration of bufalin and a shorter treatment time for both the in vitro and in vivo experiments. These results suggest a new mechanism by which low concentrations of bufalin affect TME-mediated angiogenesis; direct action on the STAT3 signalling pathway on vascular endothelial cells but not TME cells, and this effect is characterized by low toxicity and high efficiency.

In summary, our results show that the TME promotes tumour angiogenesis by activating STAT3 in vascular endothelial cells and that bufalin can precisely inhibit angiogenesis by targeting STAT3. Through our research, we have enriched the understanding of the antitumour effects of bufalin, which can indirectly inhibit TME-mediated angiogenesis. In the future, bufalin may be developed as a new type of antiangiogenic auxiliary drug.

## Conclusions

In summary, bufalin suppresses tumour microenvironment-mediated angiogenesis by inhibiting the STAT3 signalling pathway. The tumour microenvironment promotes tumour angiogenesis by activating STAT3 in vascular endothelial cells, and bufalin can precisely inhibit angiogenesis by targeting STAT3. Thus, bufalin may be used as a new antiangiogenic adjuvant therapy in the treatment of colorectal cancer.

## Supplementary Information

**Additional file 1:Figure S1.** Confirmation of CT26, CAF and TAM. a Photos of CT26 cells and CAFs. b Tumour cell and CAF marker proteins were determined using WB. c Morphological changes of TAM. d TAM determined by Flow cytometry.

**Additional file 2:Figure S2.** Effect of Plasmids transfection. HUVECs STAT3 expression was confirmed by WB (a) and quantitative PCR (b).

**Additional file 3:Figure S3.** a Body weight of subcutaneous tumor model mice. b Quantitative analysis of fluorescence intensity in subcutaneous tumor model mice. Each point represents an independent mouse.

**Additional file 4:Figure S4.** a Body weight of liver metastasis model mice. b Quantitative analysis of fluorescence intensity in liver metastasis model. Each point represents an independent mouse.

## Data Availability

The datasets during and/or analysed during the current study available from the corresponding author on reasonable request.

## Abbreviations

STAT3: Signal transducer and activator of transcription 3
TME: Tumour microenvironment
HUVECs: Human umbilical vein endothelial cells
CRC: Colorectal cancer
CAF: Cancer-associated fibroblast
TAM: Tumour-associated macrophage
BU: Bufalin
CM: Conditioned medium
WB: Western blot
PCR: Polymerase chain reaction
IHC: Immunohistochemistry
Elisa: Enzyme linked immunosorbent assay
