# Supplementary figures and images for: Bufalin suppresses tumour microenvironment-mediated angiogenesis by inhibiting the STAT3 signalling pathway
Source: J Transl Med. 2021 Sep 8;19:383. doi: 10.1186/s12967-021-03058-z (PMC8424978; doi:10.1186/s12967-021-03058-z)

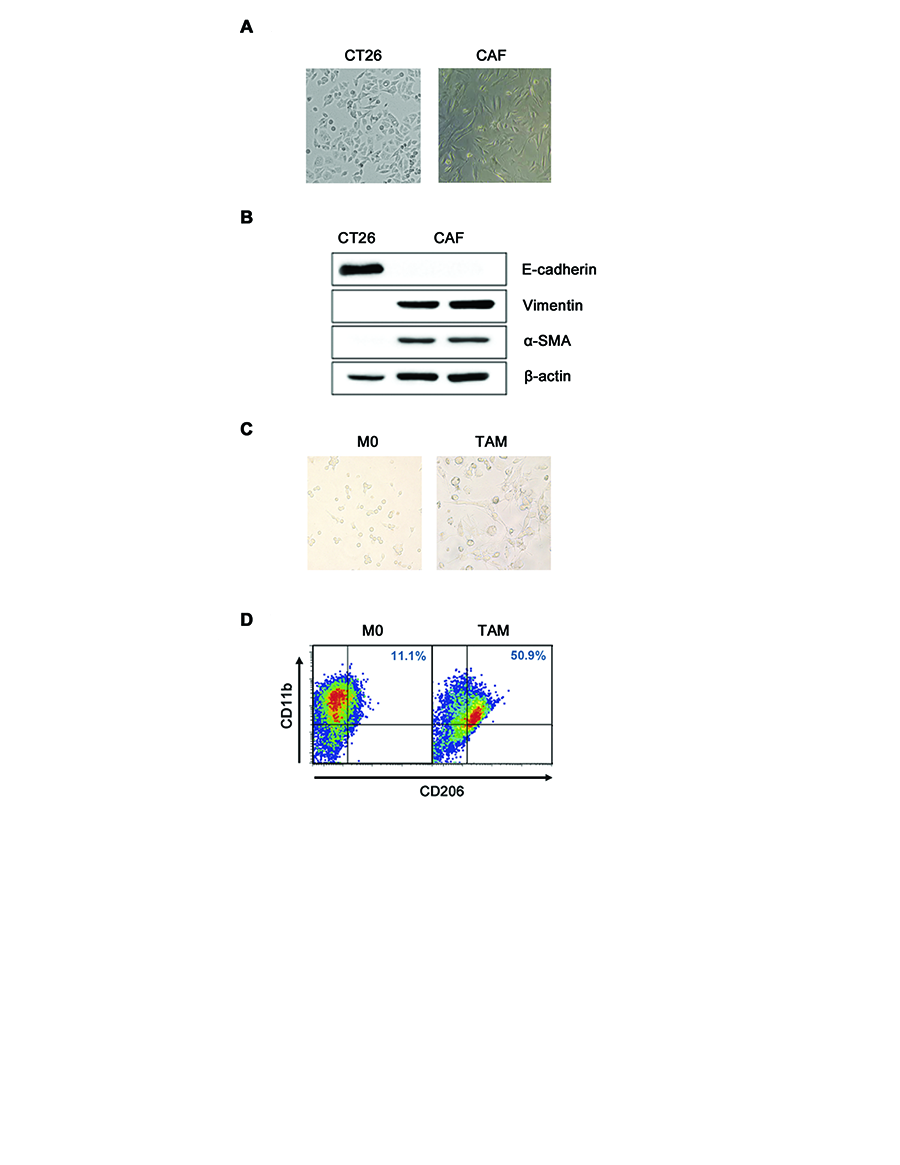

Supplement: Supplementary file 1 — Additional file 1:Figure S1. Confirmation of CT26, CAF and TAM. a Photos of CT26 cells and CAFs. b Tumour cell and CAF marker proteins were determined using WB. c Morphological changes of TAM. d TAM determined by Flow cytometry. [file 12967_2021_3058_MOESM1_ESM.tif]

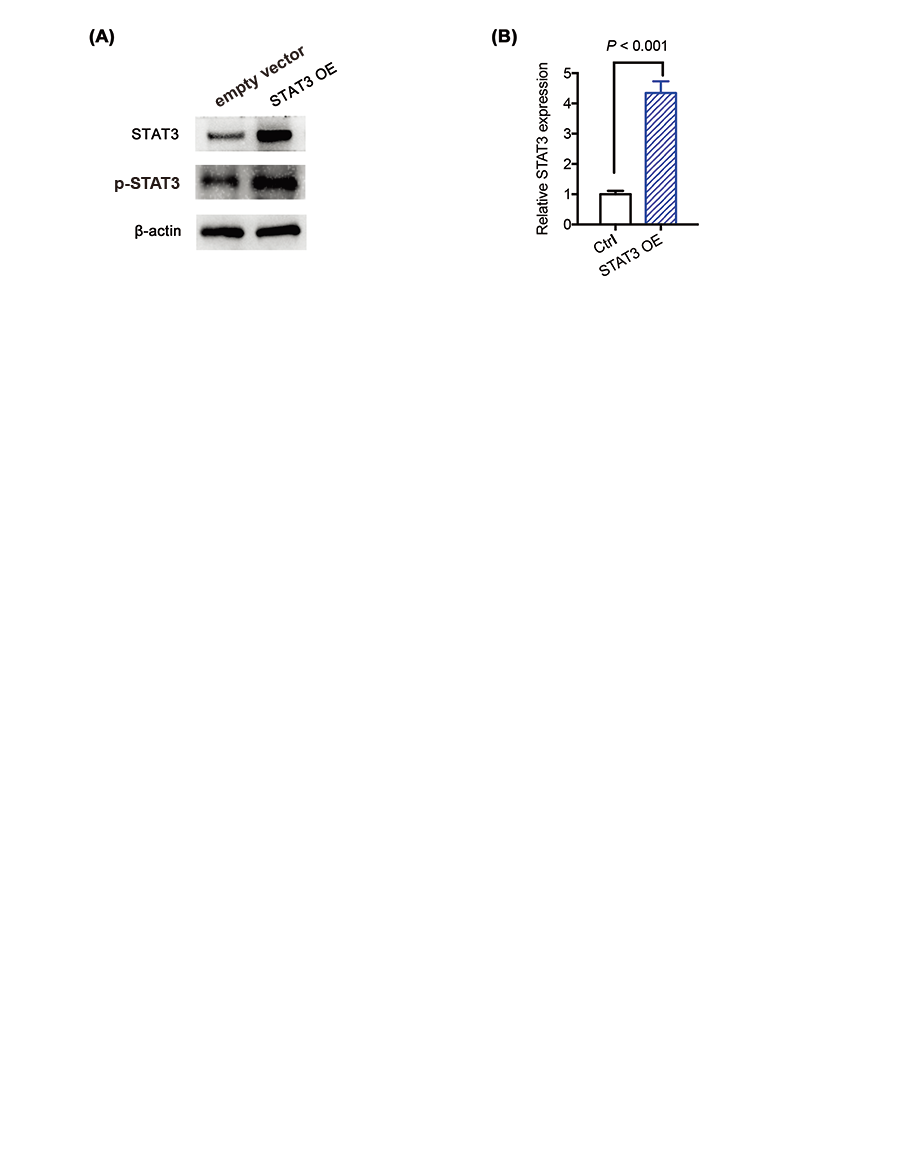

Supplement: Supplementary file 2 — Additional file 2:Figure S2. Effect of Plasmids transfection. HUVECs STAT3 expression was confirmed by WB (a) and quantitative PCR (b). [file 12967_2021_3058_MOESM2_ESM.tif]

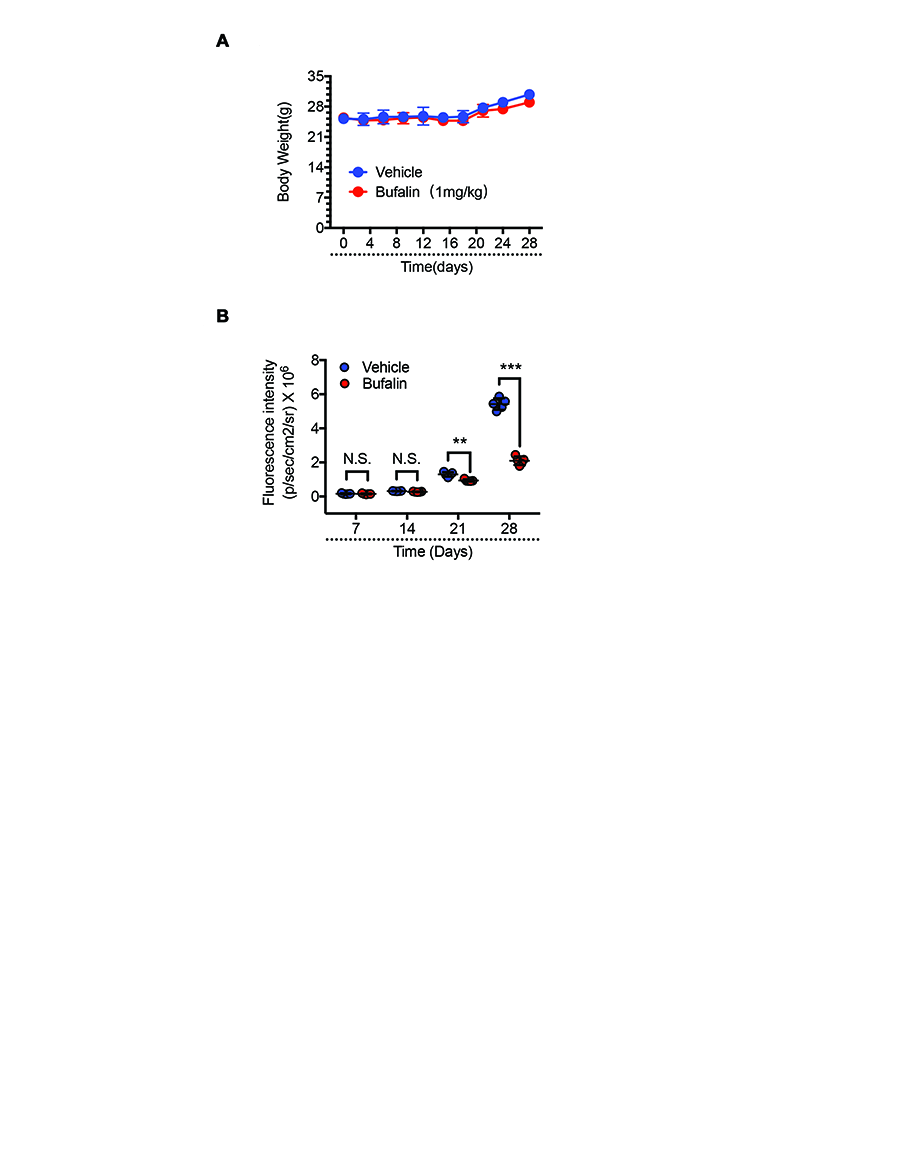

Supplement: Supplementary file 3 — Additional file 3:Figure S3. a Body weight of subcutaneous tumor model mice. b Quantitative analysis of fluorescence intensity in subcutaneous tumor model mice. Each point represents an independent mouse. [file 12967_2021_3058_MOESM3_ESM.tif]

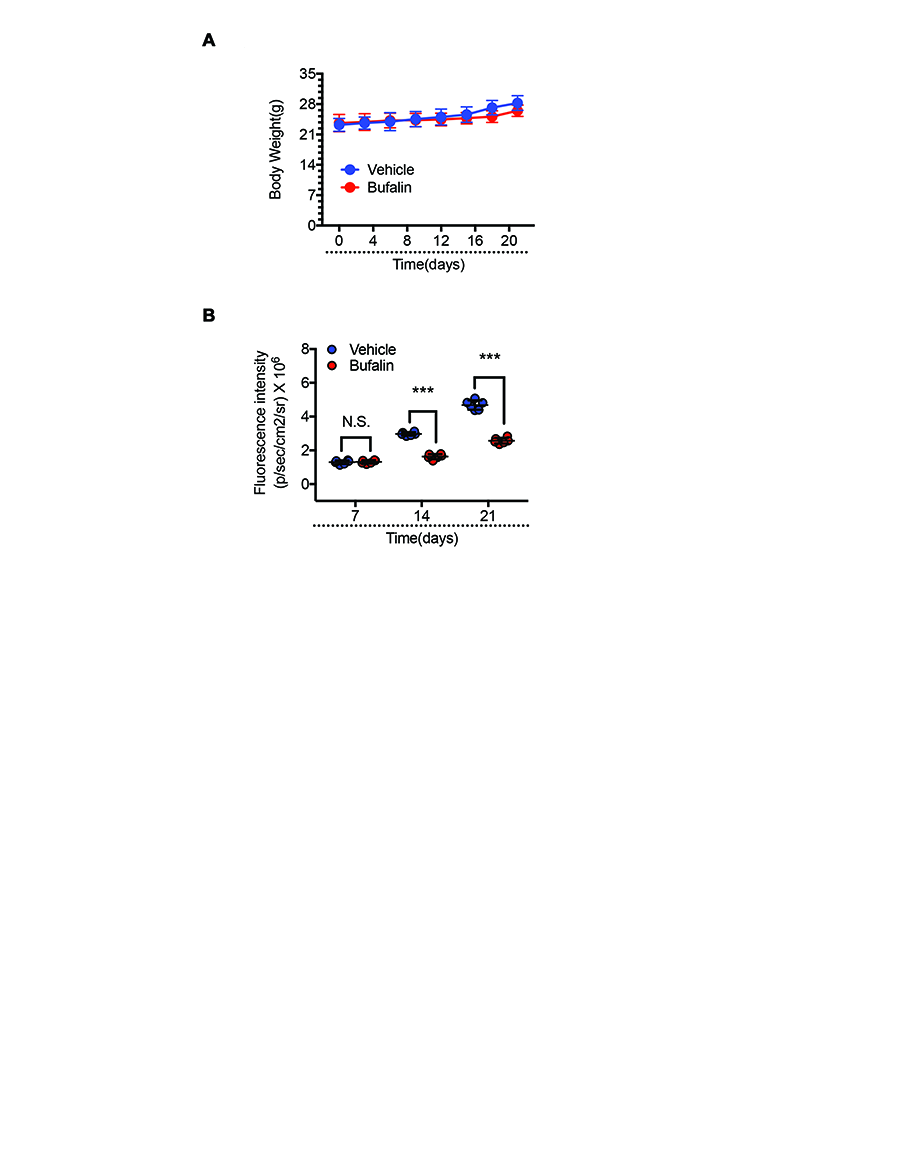

Supplement: Supplementary file 4 — Additional file 4:Figure S4. a Body weight of liver metastasis model mice. b Quantitative analysis of fluorescence intensity in liver metastasis model. Each point represents an independent mouse. [file 12967_2021_3058_MOESM4_ESM.tif]
